# Quantifying the Impacts of Pre- and Post-Conception TSH Levels on Birth Outcomes: An Examination of Different Machine Learning Models

**DOI:** 10.3389/fendo.2021.755364

**Published:** 2021-10-29

**Authors:** Yuantong Sun, Weiwei Zheng, Ling Zhang, Huijuan Zhao, Xun Li, Chao Zhang, Wuren Ma, Dajun Tian, Kun-Hsing Yu, Shuo Xiao, Liping Jin, Jing Hua

**Affiliations:** ^1^ Department of Environmental Health, Harvard T.H. Chan School of Public Health, Boston, MA, United States; ^2^ Key Laboratory of the Public Health Safety, Ministry of Education, Department of Environmental Health, School of Public Health, Fudan University, Shanghai, China; ^3^ Shanghai Ninth People’s Hospital, Shanghai Jiao Tong University School of Medicine, Shanghai, China; ^4^ Department of Epidemiology and Biostatistics, College for Public Health and Social Justice, Saint Louis University, St. Louis, MO, United States; ^5^ Department of Biomedical Informatics, Harvard Medical School, Boston, MA, United States; ^6^ Department of Pharmacology and Toxicology, Ernest Mario School of Pharmacy, Environmental and Occupational Health Sciences Institute, Rutgers University, Piscataway, NJ, United States; ^7^ Department of Maternity and Children’s Health Care Department, Shanghai First Maternity and Infant Hospital, Tongji University School of Medicine, Shanghai, China

**Keywords:** machine learning, thyroid-stimulating hormone (TSH), birth outcomes, preconception, post-conception

## Abstract

**Background:**

While previous studies identified risk factors for diverse pregnancy outcomes, traditional statistical methods had limited ability to quantify their impacts on birth outcomes precisely. We aimed to use a novel approach that applied different machine learning models to not only predict birth outcomes but systematically quantify the impacts of pre- and post-conception serum thyroid-stimulating hormone (TSH) levels and other predictive characteristics on birth outcomes.

**Methods:**

We used data from women who gave birth in Shanghai First Maternal and Infant Hospital from 2014 to 2015. We included 14,110 women with the measurement of preconception TSH in the first analysis and 3,428 out of 14,110 women with both pre- and post-conception TSH measurement in the second analysis. Synthetic Minority Over-sampling Technique (SMOTE) was applied to adjust the imbalance of outcomes. We randomly split (7:3) the data into a training set and a test set in both analyses. We compared Area Under Curve (AUC) for dichotomous outcomes and macro F1 score for categorical outcomes among four machine learning models, including logistic model, random forest model, XGBoost model, and multilayer neural network models to assess model performance. The model with the highest AUC or macro F1 score was used to quantify the importance of predictive features for adverse birth outcomes with the loss function algorithm.

**Results:**

The XGBoost model provided prominent advantages in terms of improved performance and prediction of polytomous variables. Predictive models with abnormal preconception TSH or not-well-controlled TSH, a novel indicator with pre- and post-conception TSH levels combined, provided the similar robust prediction for birth outcomes. The highest AUC of 98.7% happened in XGBoost model for predicting low Apgar score with not-well-controlled TSH adjusted. By loss function algorithm, we found that not-well-controlled TSH ranked 4^th^, 6^th^, and 7^th^ among 14 features, respectively, in predicting birthweight, induction, and preterm birth, and 3^rd^ among 19 features in predicting low Apgar score.

**Conclusions:**

Our four machine learning models offered valid predictions of birth outcomes in women during pre- and post-conception. The predictive features panel suggested the combined TSH indicator (not-well-controlled TSH) could be a potentially competitive biomarker to predict adverse birth outcomes.

## Introduction

Thyroid hormones are vital for human metabolism, energy production, early placental development, and fetal neurodevelopment (1). Thyroid-stimulating hormone (TSH) plays a crucial part in maintaining normal thyroid function, regulating the production of triiodothyronine (T3) and thyroxine (T4) (2). Thyroid function of a pregnant woman demonstrates dynamic changes as the pregnancy progresses. Stable and normal TSH during pregnancy is of significance for fetal growth, while hyperthyroidism and hypothyroidism can affect fetal birth outcomes and diseases during pregnancy (3–5).

The mechanism behind any effect of TSH levels on birth outcomes has not been revealed completely yet (6). Dysfunctional thyroxine (T4) was found associated with induced inflammation of the maternal-fetal interface, which could lead to preterm birth (7, 8). The hypothalamic–pituitary–thyroid (HPT) axis modulates the thyroid homeostasis through negative feedback, so lower T4 is strongly related to higher TSH. Abnormal TSH is, therefore, a predictor of adverse birth outcomes.

The *2017 Guidelines of the American Thyroid Association (ATA) for the Diagnosis and Management of Thyroid Disease During Pregnancy and the Postpartum* referred a TSH of 2.5 mIU/L as the upper limit for TSH control in the first trimester (9). In the National Pre-pregnancy Checkups Project during 2010 to 2012, mothers with TSH level of 2.50–4.29 mIU/L prior to conception were more likely to have a preterm birth than those with TSH level of 0.48–2.50 mIU/L (10). However, Khan et al.’s study indicated that the level of TSH exceeding 2.5 mIU/L in both pre- and post-conception had no association with birth outcomes (11). Therefore, researches regarding the association between TSH levels (>2.5 mIU/L) and birth outcomes have met no consensus yet.

The guidelines of ATA recommended a normal lower limit reference of TSH of 0.10 mIU/L for Chinese women in early pregnancy (9). However, there was limited literature available that investigated the relationship between TSH levels being less than 0.10 mIU/L or combined two TSH measurements during pre- and post-conception on birth outcomes.

In recent years, research in machine learning and deep learning has undergone tremendous growth and has achieved compelling new results in the realm of medical image recognition (12, 13). Nonetheless, studies implementing machine learning and deep learning techniques to predict the birth outcomes based on TSH levels are scarce. While traditional studies have identified risk factors for diverse pregnancy outcomes such as premature birth and birthweight, they have not been able to quantify their impacts or feature importance on predicting these birth outcomes accurately (14).

To address this gap, our study used a novel approach that applied the loss function algorithm in machine learning to systematically quantify the impacts of pre- and post-conception serum TSH levels and other risk factors on four birth outcomes: induction, preterm birth, neonatal Apgar score, and birthweight.

## Methods

### Study Design and Participants

We used data from women who gave birth in Shanghai First Maternal and Infant Hospital from April 1 2014 to December 31 2015. We analyzed the data in two different ways. In the first way of analysis, we excluded women without preconception TSH, complete predictive variables (such as gravidity and parity) or birth outcomes, and 14,110 pregnant women were included finally. The second way of analysis included 3,428 subjects from the first analysis who also had TSH data in the first trimester. The study protocol was approved by the ethics committee of the Shanghai First Maternity and Infant Hospital, Tongji University School of Medicine.

### Data Handling

Maternal characteristics in the data from each observation were included in the analysis. Predictive characteristics included (1) subjects’ demographic features such as age, occupation, and ethnicity (2); previous pregnancy history such as gravidity, parity, and cesarean scar uterus (3); symptoms and comorbidities such as gestational diabetes, gestational hypertension, preeclampsia, fever, renal disease, and placenta previa (4); number of fetus (5); pre- and post-conception TSH. The outcome characteristics included neonatal Apgar scores (0–10), neonatal birthweight (g), gestational age (week), and labor induction. We used one-hot encoding to convert the polytomous variables among the predictive variables into dummy variables in order to facilitate machine identification. For the dependent variables, we converted them into dummy or categorical variables: preterm birth (gestational age <37 weeks and >=28 weeks) *vs* full-term delivery (gestational age >= 37 weeks), neonatal low Apgar scores (<7) *vs* normal Apgar Score (>=7), and neonatal birthweight (low birthweight for <2,500g, normal for 2,500–4,000 g, and macrosomia for >4,000 g). According to the Chinese Perinatal Medical Association, TSH in early pregnancy was recommended to be controlled within 0.1–2.5 mIU/L (15); hence, preconception TSH levels lower than 0.1 mIU/L or higher than 2.5 mIU/L was recognized as abnormal preconception TSH levels. In the second analysis, we defined well-controlled TSH if both pre- and post-conception TSH levels were 0.1–2.5 mIU/L. Either pre- or post-conception TSH beyond that range was considered as not-well-controlled TSH. Post-conception TSH level referred to the first TSH level measured after conception in early pregnancy.

The outcome variables showed class imbalance at large; for instance, among the 14,110 pregnant women included in the first analysis, only 5.9%, 835 subjects, had a preterm birth. We used Synthetic Minority Over-sampling Technique (SMOTE) to balance the categorical data (16). After adjusting the class balance, we divided the synthetic data into a training set and a test set (70%:30% split). The model performance in the test set was reported and compared.

### Statistical Analysis and Machine Learning Approach

We used various machine learning predictive models, including logistic model, random forest model, XGBoost model, and deep learning neural network models to predict the birth outcomes. Eighteen converted dummy predictive variables including demographic features, previous pregnancy history, symptoms and comorbidities, number of fetus, and TSH conditions were adjusted for each model. In the first analysis, we included abnormal preconception TSH as a predictive feature. In the second analysis, we included abnormal preconception TSH or not-well-controlled TSH as a predictive feature. We established the confusion matrix of the test set and calculated the accuracy, precision, recall, F1 score, and AUC for test set in each model. Then the model with the best performance—highest AUC for dichotomous outcomes or macro F1 score for categorical outcomes—was selected to sort each feature’s importance with the loss function algorithm.

The structure of the multilayer neural network model consisted of six layers (three dense layers with relu activation function, one dropout layer with 10 or 20% dropout rate, one flatten layer, and one output layer). The parameters used to build the model were optimized using stochastic gradient descent (SGD) algorithm using a batch size of 128.

Whereas many models came with the algorithms that calculated feature importance, most of them were complex and rarely represented the significance of a feature directly. In this study, the indicator “percentage increase of loss (%incloss)” after a feature removal was employed to describe the importance of a feature (variable). The loss function for dichotomous and polytomous outcome variables is cross entropy (17), and the base for logarithm calculation is 2.

The calculation formula of cross entropy for the dichotomous outcome variables is


$$\frac{-1}{n}\sum_{i=1}^{n}\left(y_{i}*\log(p_{i})+(1-y_{i})*\log(1-p_{i})\right)$$

where *y_i_* is the outcome variable, *p_i_* is the predicted probability of outcomes, and *n* is the number of outcomes.

The calculation formula of cross entropy for the polytomous outcome variables is


$$\frac{1}{n}\sum_{i=1}^{n}\sum_{c=1}^{C}y_{i,c}*\log p_{i,c}$$

where *y_i_* is the outcome variable, *p_i_* is the predicted probability of outcomes, *n* is the number of outcomes, and *c* is the number of categories.

The calculation formula of the percentage increase of loss is


$$\%\text{incloss}=\frac{loss_{j-1}-loss_{j}}{loss_{j}}*100\%$$

where *j* is the number of feature variables, *loss_j_* is the model loss before feature removals, and *loss_j-1_* is the model loss after a feature removal.

If %incloss>0, the feature is considered necessary for model prediction; if %incloss=0, this feature is considered not affecting the model prediction; if %incloss<0, this feature is considered unnecessary for model prediction and the performance of the model would improve after removing this feature.

We did all analyses using Python (version 3.7.3).

## Results

Predictive characteristics including pre- and post-conception TSH levels for subjects in the first and second analyses were shown (**Table 1**). More than 98% enrolled women were under 40 years, and the age structures for both analyses were very similar (p=0.19). Most women were of Han ethnicity. More than half of the enrolled women had single gravidity as well as parity. The proportion of enrolled women who had cesarean scar uterus was significantly higher in the first analysis (p<0.01). The mean of preconception TSH in the second analysis was 0.41 mIU/L higher than the mean of that in the first analysis (p<0.01), and the proportion of enrolled women with abnormal preconception TSH was also significantly higher in the second analysis (p<0.01). According to our definition, 50.1% of enrolled women in the second analysis were considered having not-well-controlled TSH.

**Table 1:** Baseline predictive characteristics for subjects in the first and second analysis.

Demographics	Subjects in the first analysis (N = 14,110)	Subjects in the second analysis (N = 3,428)	P value
Age group n(%)			0.19
<30	6,544 (46.4%)	1,584 (46.2%)	
30–39	7,339 (52.0%)	1,803 (52.6%)	
>=40	227 (1.6%)	41 (1.2%)	
Ethnicity n(%)			<0.01
Han	13,904 (98.5%)	3,352 (97.8%)	
Others	206 (1.5%)	76 (2.2%)	
Occupation n(%)			<0.01
Company staff	11,566 (82.0%)	2,714 (79.2%)	
Other occupations	1,625 (11.5%)	455 (13.3%)	
Unemployed	919 (6.5%)	259 (7.5%)	
Gravidity n(%)			0.04
1	7,584 (53.8%)	1,776 (51.8%)	
>1	6,526 (46.2%)	1,652 (48.2%)	
Parity n(%)			<0.01
1	11,165 (79.1%)	2,882 (84.1%)	
>1	2,945 (20.9%)	546 (15.9%)	
Cesarean scar n(%)	1,499 (10.6%)	293 (8.5%)	<0.01
Gestational diabetes n(%)	1,946 (13.8%)	492 (14.4%)	0.41
Gestational hypertension n(%)	432 (3.1%)	108 (3.2%)	0.83
Preeclampsia n(%)	164 (1.2%)	45 (1.3%)	0.52
Fever n (%)	1,058 (7.5%)	305 (8.9%)	<0.01
Renal disease n(%)	80 (0.6%)	20 (0.6%)	1.00
Placenta previa n(%)	108 (0.8%)	24 (0.7%)	0.77
Number of fetus n(%)			0.30
1	13,722 (97.3%)	3,322 (96.9%)	
>1	388 (2.7%)	106 (3.1%)	
TSH (mIU/L) mean (SD)			
Preconception TSH	1.68 (1.66)	2.09 (2.80)	<0.01
Post-conception TSH		1.82 (1.77)	
Abnormal preconception TSH n(%)	3,198 (22.7%)	1,472 (42.9%)	<0.01
Not-well-controlled TSH n(%)		1,717 (50.1%)	

*TSH, thyroid stimulating hormone.

We also incorporated tables with both predictive characteristics and outcomes for the first and second analyses (**Supplementary Tables 1** and **2**). We found an ascending trend for maternal preconception TSH with the birthweight increasing in both analyses. Compared with those with normal neonatal Apgar score, enrolled women with low neonatal Apgar score had higher mean preconception TSH or/and post-conception TSH in both analyses. Subjects with induction had higher odds of fever than those without induction in both analyses.

In the first analysis, the XGBoost model showed the best performance with the highest AUC compared to others except predicting induction, and it also displayed better performance in predicting low Apgar scores (<7) and birthweight with multiple categories (**Table 2**). However, the overall model performance for predicting four birth outcomes was not ideal. Four models were not very capable of distinguishing three birthweight categories. Although the AUC for predicting induction was no more than 60%, the recall rate that measures the ability to identify the true positives could be as high as 86.3% for predicting induction (**Supplementary Table 3**). Multilayer neural network showed model performance no better than the logistic model.

**Table 2:** Model performance of synthetic data from subjects in the first analysis.

Outcome variables	Preterm Birth	Low Apgar Score^a^	Birthweight^b^	Induction
Logistic model	64.2%	75.8%	47.6%	59.5%
Random forest model	65.5%	77.0%	47.8%	59.9%
XGBoost model	65.8%	80.9%	50.7%	59.5%
Multilayer neural network	63.9%	75.0%	42.7%	59.3%

*18 dummy predictive features were adjusted in four models.

^a^Five more variables on delivery process were adjusted in the predictive model of low Apgar score. The five extra variables were fetal position, neonatal injury during delivery, delivery method, lateral episiotomy, and vaginal midwifery.

^b^Model performance of birthweight was assessed with macro F1 score instead of AUC.

**Figure 1:**
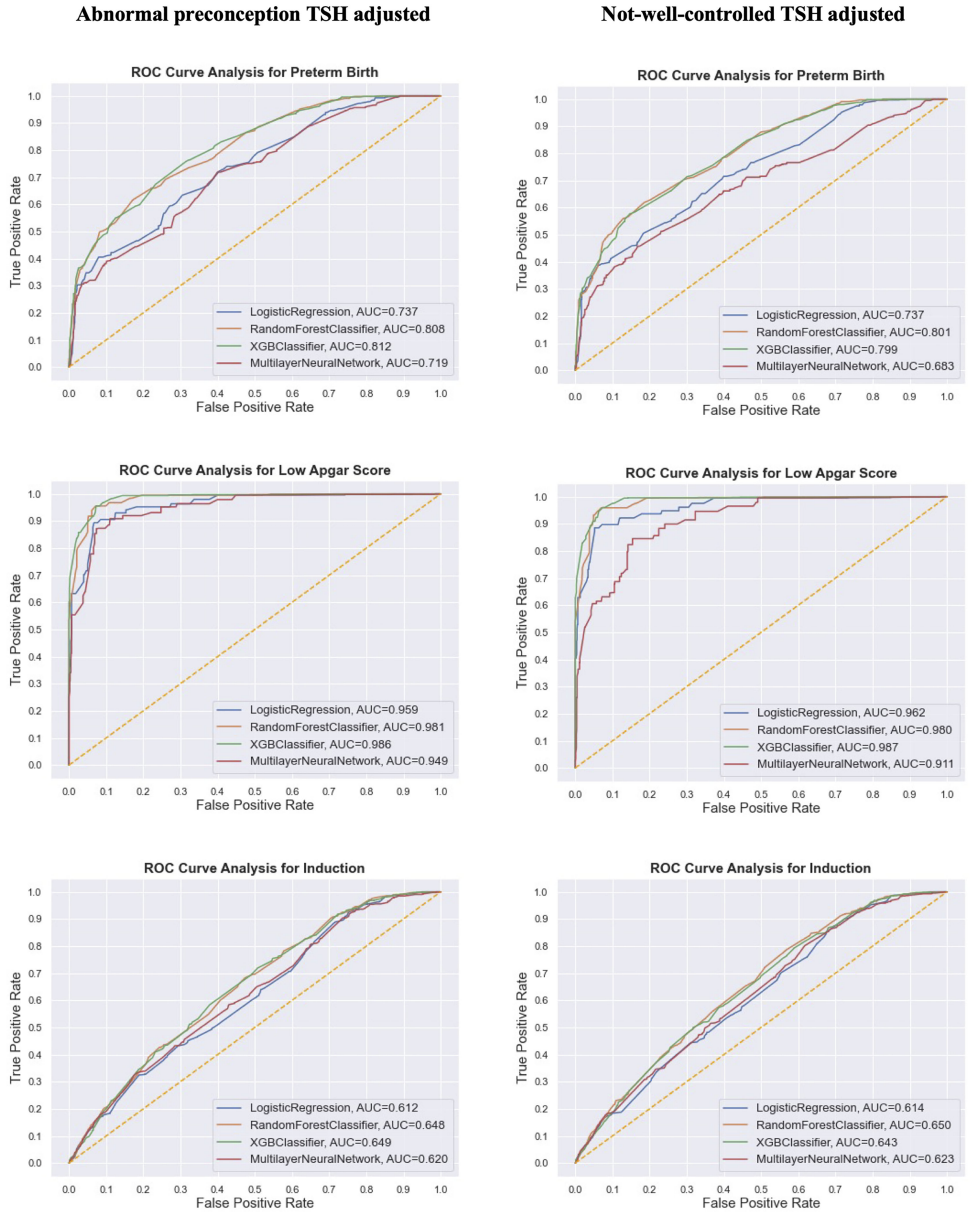
Comparison of model performance (AUC/ROC curve) of the synthetic data on three dichotomous birth outcomes from the research subjects in the second analysis. Different TSH derivatives were adjusted in two separate predictive models with other predictive features staying the same.

In the second analysis, we then compared the AUC (or macro F1 score) of the prediction of birth outcomes adjusted for two different derivatives from pre and post TSH levels (1): abnormal preconception TSH (2), not-well-controlled TSH (**Figure 1** for dichotomous outcomes and **Supplementary Table 4** for categorical outcomes). When keeping other predictive characteristics the same, the AUC showed no significant difference between both TSH scenarios. The highest AUC of 98.7% happened when XGBoost model predicted low Apgar score with not-well-controlled TSH. Although the number of subjects in the second analysis was much fewer than that in the first analysis, the models in the second analysis performed much better than those in the first analysis when predicting the same birth outcomes. But the four models were still not capable of predicting outcomes with multiple categories like birthweight.

**Figure 2:**
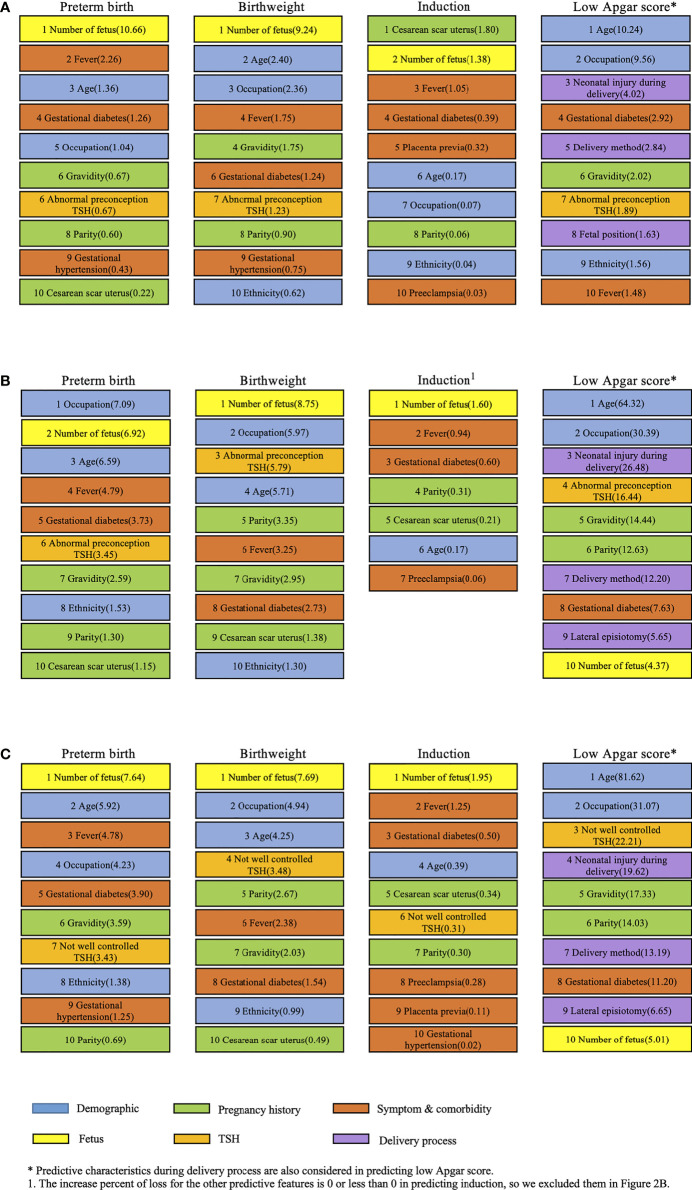
Leading predictive features (%incloss) for four birth outcomes in both analyses of subjects. **(A)** Leading predictive features for four birth outcomes among women in the first analysis with abnormal preconception TSH based on the XGBoost model. **(B)** Leading predictive features for four birth outcomes among women in the second analysis with abnormal preconception TSH based on the XGBoost model. **(C)** Leading predictive features for four birth outcomes among women in the second analysis with not-well-controlled TSH based on the XGBoost model.

Since XGBoost model showed more power of predicting birth outcomes than others, it was chosen to calculate the importance of each predictive feature with loss function. **Figure 2** presents the leading predictive features for birth outcomes among women in the first (**Figure 2A**) and second (**Figures 2B, C**) analysis. In the first analysis, we found that the leading predictive features were quite different across four birth outcomes. Number of fetus was the most important feature to predict preterm birth and birthweight, while cesarean scar uterus impacted the induction most. Age was the top predictive feature for low Apgar score for which five more features on delivery process were also adjusted. Besides age, maternal occupation and neonatal injury during delivery were the prominent predictive features of low Apgar score for newborns. Three out of five features during delivery process were among the top 10 predictive features to predict low Apgar score. Besides, abnormal preconception TSH served as a moderate predictive characteristic for preterm birth, birthweight, and low Apgar score in the first analysis.

The number of fetus was also the most important feature in predicting three birth outcomes except low Apgar score in the second analysis. The derivatives of TSH were different in **Figures 2B, C** when the other predictive features stayed the same in calculating the feature importance with loss function. The patterns of predictive feature ranking in **Figures 2B, C** were much similar than **Figures 2A, B**. Abnormal preconception TSH ranked 6^th^, 3^rd^, and 4^th^ in predicting preterm birth, birthweight, and low Apgar score, respectively. Meanwhile, not-well-controlled TSH ranked 7^th^, 4^th^, and 3^rd^ to predict these three birth outcomes. Surprisingly, not-well-controlled TSH ranked 6^th^ in predicting induction, while abnormal preconception TSH did not show the capability of predicting induction in **Figures 2A, B**.

## Discussion

The research subjects of this study were analyzed in two different ways. Machine learning models were implemented to predict four birth outcomes. Overall, XGBoost model showed outstanding performance in predicting birth outcomes. With respect to the predictive characteristics ranking, the leading predictive features of different outcomes revealed great diversity in both analyses of the study.

For the first analysis of the study, XGBoost model displayed higher performance for polytomous variables (birthweight) than that of other models. It was also indicated in **Table 2** that the performance of the neural network model was not significantly better than other models, and it was even no better than logistic model. Moreover, since the neural network model has high randomness (18), we have to set random seed to ensure reproducibility and obtain the same results. Engchuan et al. applied the loss function algorithm with continuous variables in the research on social determinants of health, and they used the mean square error percentage increase to measure the importance of a feature variable (19). However, we employed the loss function algorithm for dichotomous and categorical variables in this study.

The difference in predictive characteristics ranking between both analyses of subjects with abnormal preconception TSH could be attributed to the difference in underlying conditions of two groups of subjects. The subjects in the second analysis were the subgroup of the subjects in the first analysis. Although subjects in both analyses shared the similar age structure, the levels of preconception TSH in the second analysis were significantly higher than that in the first analysis. Abnormal preconception TSH and not-well-controlled TSH showed similar rankings to predict birth outcomes, while the latter had potential to predict induction. Although the mechanism behind this was not clear, the combined TSH indicator could be a competitive biomarker for maternal health.

We found that fever served as an important symptom feature for birth outcome prediction, especially in predicting preterm birth and induction in both analyses. Abnormal preconception TSH ranked 6^th^ out of 14 in both analyses and showed the capability of predicting preterm birth. Also, it had the third place in the prediction of birthweight in the second analysis. Therefore, it can be concluded that the importance of preconception TSH levels in predicting adverse birth outcomes was beyond average level. This finding was consistent with the existing literature on the relationship between pre-pregnancy TSH and birth outcomes, such as preterm birth. Chen et al. introduced 2.5 mIU/L as a cutoff for preconception TSH levels in their study, where they found that compared with mothers with a TSH level between 0.48 and 2.50 mIU/L, those with a TSH level of 2.50–4.29 mIU/L prior to conception had a higher risk of preterm birth (OR:1.09, 95% CI: 1.04–1.15) (10). Furthermore, another study suggested maternal serum TSH concentration >4 mIU/L in pregnancy was associated with approximately twofold increased risks of preterm birth and elevated TSH was also associated with increases in the risk of preeclampsia and low birth weight (20). A cohort study found inverse association between maternal TSH in mid-gestation and neonatal birthweight (21). Therefore, abnormal TSH across different trimesters was proved to be associated with adverse birth outcomes in a variety of studies.

In the second analysis, we defined either pre- or post-conception TSH beyond 0.1–2.5 mIU/L was considered as not-well-controlled TSH, which indicated a higher risk of thyroid dysfunctions (hyperthyroidism or hypothyroidism). Although some papers may argue hyperthyroidism is defined as suppressed (usually undetectable) thyrotropin (TSH) and elevated levels of triiodothyronine (T3) and/or estimated free thyroxine (free T4) (22, 23), we used both measurements of TSH during pre- and post-conception beyond normal range by the Chinese guideline (15) as a strong indicator of thyroid dysfunction regardless of T3 and T4. Since few previous studies included the two TSH levels for discussion, this paper employed the two TSH measurements together to analyze the influence of TSH changes on four birth outcomes.

Many studies have discussed the effects of hypothyroidism pre- and post-pregnancy on the maternal and offspring’s health outcomes. Kiran et al. (24) found that women with hypothyroidism diagnosed before conception had significantly higher risk of neonatal low birthweight. Also a prospective cohort study not only concluded the odds ratio of neonatal low birthweight in women with maternal hypothyroidism (IMH) was 2.53-folder higher than the healthy women, but also demonstrated the women with IMH had 5.43 times higher risk of preterm premature rupture of the membranes (PPROM) (25). Other studies showed concrete evidence on the association of subclinical hypothyroidism with the adverse outcomes in both short and long term, such as higher risk of pregnancy loss (26), offspring’s behavioral alterations (27). Whereas scientific studies on gestational hypothyroidism are abundant, there are not many studies on the influence of TSH below 0.1 mIU/L, which may be an indicator of higher risk of hyperthyroidism, concerning birth outcomes. Our study evaluated the overall impacts of abnormal TSH levels higher than 2.5 mIU/L and lower than 0.1 mIU/L on birth outcomes. Although we incorporated pre- and post-conception TSH to create a novel indicator, the health effect of TSH lower than normal during early pregnancy on neonatal outcomes remains unknown. To reveal the effects of low TSH on the maternal and offspring’s health outcomes, the scientific community requires more literature and methods, including traditional epidemiological studies as well as machine learning tools.

However, there were still some limitations in this study. First, the study did not consider incorporating FT4 and anti-thyroid antibodies in the prediction of birth outcomes. The recently published paper implied that FT4 had more impacts on birth outcomes (like birthweight) than TSH did (28). TSH might not have direct association with pregnancy outcomes. Therefore, more studies to reveal the roles of FT4 on predicting birth outcomes are needed in the future. Second, selection bias could potentially affect the association of interest. Patients enrolled might have potential thyroid issues, so the TSH levels in our study may not reflect the thyroid functions of general people. Nevertheless, thyroid function screening is becoming a prevalent measurement in maternal and infant clinics in Shanghai. And selection bias would be minimized in the near future. Though educational and lifestyle factors (workout frequency and others) were not considered, this paper incorporated a variety of pregnant symptoms and comorbidities, such as gestational diabetes, gestational hypertension, preeclampsia, fever, and kidney disease. Besides demographic factors, the health conditions of mothers also have an impact on birth outcomes. Demographic factors may influence the birth outcomes through the health conditions of mothers. In terms of alcohol consumption and oral contraceptives intake, Li et al.’s cross-sectional study found that 98.1% of pregnancies did not drink alcohol during pregnancy (29); Cheng Li et al.’s case-control study on five medical centers in Shanghai between 2011 and 2013 demonstrated that only 1.42% of normal pregnant women had used oral contraceptives (30). Therefore, alcohol consumption and oral contraceptive intake has been ignored in this study.

In conclusion, we examined four machine learning models to predict birth outcomes, namely, preterm birth, birthweight, low Apgar score, and induction. The XGBoost model provided the most robust prediction overall and performed better on categorical outcomes. We also created a novel indicator, not-well-controlled TSH, by combining pre- and post-conception TSH. The novel indicator performed well, and the highest AUC of 98.7% happened in XGBoost model for predicting low Apgar score with this TSH indicator. The predictive panel suggested not-well-controlled TSH had top ranks in predicting specific adverse birth outcomes. This competitive biomarker and machine learning tools are expected to be more widely used in the realm of precisely perinatal medicine. More novel indicators from thyroid hormones including T4 and anti-thyroid antibody are waiting to be excavated. And the quantifications of predictive features on maternal and offspring’s health will be thriving in future studies.

## Data Availability Statement

The data analyzed in this study is subject to the following licenses/restrictions: The application to the dataset should be approved by the Shanghai First Maternity and Infant Hospital. Requests to access these datasets should be directed to jinlp01@163.com.

## Ethics Statement

The studies involving human participants were reviewed and approved by the Ethics Committee of Shanghai First Maternity and Infant Hospital. Written informed consent to participate in this study was provided by the participants’ legal guardian/next of kin.

## Author Contributions

YS: study conception and design; project administration; data collection; data analysis; writing—original draft preparation. WZ: study conception and design; project administration; data collection; data analysis; writing—original draft preparation. LZ: data analysis. HZ: data collection and cleaning. XL: data collection and cleaning. CZ: model validation. WM: model validation. DT: data analysis and checking. K-HY: writing—review and editing. SX: manuscript review and revision. LJ: study conception and design; project administration. JH: study conception and design; project administration. All authors contributed to the article and approved the submitted version.

## Funding

This work was supported by the National Natural Science Foundation of China (Nos. 81773379, 82122058, and 81202165), the Shanghai Municipal Commission of Health and the Family Foundation for Fifth Round of the Three-Year Public Health Action Plan of Shanghai (GWV-1.1 and GWV-10.1-XK08 and 202040186), and for Young Talents (2017YQ023). K-HY was supported by the Harvard Data Science Fellowship. Natural Science Foundation of Shanghai (21 ZR 1438400).

## Conflict of Interest

The authors declare that the research was conducted in the absence of any commercial or financial relationships that could be construed as a potential conflict of interest.

## Publisher’s Note

All claims expressed in this article are solely those of the authors and do not necessarily represent those of their affiliated organizations, or those of the publisher, the editors and the reviewers. Any product that may be evaluated in this article, or claim that may be made by its manufacturer, is not guaranteed or endorsed by the publisher.
