# Supplementary material for: Quantifying the Impacts of Pre- and Post-Conception TSH Levels on Birth Outcomes: An Examination of Different Machine Learning Models
Source: Front Endocrinol (Lausanne). 2021 Oct 29;12:755364. doi: 10.3389/fendo.2021.755364 (PMC8586450; doi:10.3389/fendo.2021.755364)
Supplement: Supplementary file 1 [file Table_1.docx]

**Supplementary Table 1 Predictive characteristics and birth outcomes for subjects in the first analysis**

| Predictive characteristics | Subjects in the first analysis  (N=14110) | | | | | | | | |
| --- | --- | --- | --- | --- | --- | --- | --- | --- | --- |
|  | Preterm birth | | Apgar score | | Birthweight | | | Induction | |
|  | Yes  (N=835) | No  (N=13275) | < 7  (N=106) | >=7  (N=14004) | Low birthweight  (N=556) | Normal  (N=12770) | Macrosomia  (N=784) | Yes  (N=3958) | No  (N=10152) |
| Age n(%) |  |  |  |  |  |  |  |  |  |
| < 30 | 328  (39.3%) | 6216  (46.8%) | 41  (38.7%) | 6503  (46.4%) | 271  (39.0%) | 5991  (46.9%) | 336  (42.9%) | 2087  (52.7%) | 4457  (43.9%) |
| 30-39 | 487  (58.3%) | 6852  (51.6%) | 62  (58.5%) | 7277  (52.0%) | 331  (59.5%) | 6574  (51.5%) | 434  (55.4%) | 1834  (46.3%) | 5505  (54.2%) |
| >=40 | 20  (2.4%) | 207  (1.6%) | 3  (2.8%) | 224  (1.6%) | 8  (1.4%) | 205  (1.6%) | 14  (1.8%) | 37  (0.9%) | 190  (1.9%) |
| Ethnicity n(%) |  |  |  |  |  |  |  |  |  |
| Han | 826  (98.9%) | 13078  (98.5%) | 104  (98.1%) | 13800  (98.5%) | 546  (98.2%) | 12587  (98.6%) | 771  (98.3%) | 3896  (98.4%) | 10008  (98.6%) |
| Others | 9  (1.1%) | 197  (1.5%) | 2  (1.9%) | 204  (1.5%) | 10  (1.8%) | 183  (1.4%) | 13  (1.7%) | 62  (1.6%) | 144  (1.4%) |
| Occupation n(%) |  |  |  |  |  |  |  |  |  |
| Company staff | 688  (82.4%) | 10878  (81.9%) | 92  (86.8%) | 11474  (81.9%) | 469  (84.4%) | 10452  (81.8%) | 645  (82.3%) | 3293  (83.2%) | 8273  (81.5%) |
| Other occupations | 81  (9.7%) | 1544  (11.6%) | 10  (9.4%) | 1615  (11.5%) | 49  (8.8%) | 1497  (11.7%) | 79  (10.1%) | 445  (11.2%) | 1180  (11.6%) |
| Unemployed | 66  (7.9%) | 853  (6.4%) | 4  (3.8%) | 915  (6.5%) | 38  (6.8%) | 821  (6.4%) | 60  (7.7%) | 220  (5.6%) | 699  (6.9%) |
| Gravidity n(%) |  |  |  |  |  |  |  |  |  |
| 1 | 416  (49.8%) | 7168  (54.0%) | 64  (60.4%) | 7520  (53.7%) | 307  (55.2%) | 6920  (54.2%) | 357  (45.5%) | 2433  (61.5%) | 5151  (50.7%) |
| >1 | 419  (50.2%) | 6107  (46.0%) | 42  (39.6%) | 6484  (46.3%) | 249  (44.8%) | 5850  (45.8%) | 427  (54.5%) | 1525  (38.5%) | 5001  (49.3%) |
| Parity n(%) |  |  |  |  |  |  |  |  |  |
| 1 | 627  (75.1%) | 10538  (79.4%) | 91  (85.8%) | 11074  (79.1%) | 444  (79.9%) | 10139  (79.4%) | 582  (74.2%) | 3536  (89.3%) | 7629  (75.1%) |
| >1 | 208  (24.9%) | 2737  (20.6%) | 15  (14.2%) | 2930  (20.9%) | 112  (20.1%) | 2631  (20.6%) | 202  (25.8%) | 422  (10.7%) | 2523  (24.9%) |
| Cesarean scar uterus n(%) | 107  (12.8%) | 1392  (10.5%) | 7  (6.6%) | 1492  (10.7%) | 54  (9.7%) | 1355  (10.6%) | 90  (11.5%) | 108  (2.7%) | 1391  (13.7%) |
| Gestational diabetes n(%) | 181  (21.7%) | 1765  (13.3%) | 13  (12.3%) | 1933  (13.8%) | 103  (18.5%) | 1702  (13.3%) | 141  (18.0%) | 605  (15.3%) | 1341  (13.2%) |
| Gestational hypertension n(%) | 33  (4.0%) | 399  (3.0%) | 5  (4.7%) | 427  (3.0%) | 23  (4.1%) | 387  (3.0%) | 22  (2.8%) | 157  (4.0%) | 275  (2.7%) |
| Preeclampsia n(%) | 19  (2.3%) | 145  (1.1%) | 1  (0.9%) | 163  (1.2%) | 16  (2.9%) | 141  (1.1%) | 7  (0.9%) | 65  (1.6%) | 99  (1.0%) |
| Fever n(%) | 23  (2.8%) | 1035  (7.8%) | 18  (17.0%) | 1040  (7.4%) | 14  (2.5%) | 979  (7.7%) | 65  (8.3%) | 557  (14.1%) | 501  (4.9%) |
| Renal disease n(%) | 4  (0.5%) | 76  (0.6%) | 0  (0%) | 80  (0.6%) | 3  (0.5%) | 72  (0.6%) | 5  (0.6%) | 24  (0.6%) | 56  (0.6%) |
| Placenta previa n(%) | 37  (4.4%) | 71  (0.5%) | 0  (0%) | 108  (0.8%) | 21  (3.8%) | 85  (0.7%) | 2  (0.3%) | 4  (0.1%) | 104  (1.0%) |
| Number of fetus n(%) |  |  |  |  |  |  |  |  |  |
| 1 | 607  (72.7%) | 13115  (98.8%) | 103  (97.2%) | 13619  (97.3%) | 376  (67.6%) | 12562  (98.4%) | 784  (100%) | 3953  (99.9%) | 9769  (96.2%) |
| >1 | 228  (27.3%) | 160  (1.2%) | 3  (2.8%) | 385  (2.7%) | 180  (32.4%) | 208  (1.6%) | 0  (0%) | 5  (0.1%) | 383  (3.8%) |
| TSH (mlU/L) mean(SD) |  |  |  |  |  |  |  |  |  |
| Preconception | 1.58  (1.17) | 1.68  (1.68) | 1.89  (1.63) | 1.68  (1.66) | 1.57  (1.20) | 1.67  (1.30) | 1.82  (4.57) | 1.72  (1.51) | 1.66  (1.71) |
| Post-conception |  | | | | | | | | |
| Abnormal preconception TSH n(%) | 217  (26.0%) | 2981  (22.5%) | 31  (29.2%) | 3167  (22.6%) | 152  (27.3%) | 2873  (22.5%) | 173  (22.1%) | 937  (23.7%) | 2261  (22.3%) |
| Not well controlled TSH n(%) |  | | | | | | | | |
