# Supplementary material for: Quantifying the Impacts of Pre- and Post-Conception TSH Levels on Birth Outcomes: An Examination of Different Machine Learning Models
Source: Front Endocrinol (Lausanne). 2021 Oct 29;12:755364. doi: 10.3389/fendo.2021.755364 (PMC8586450; doi:10.3389/fendo.2021.755364)
Supplement: Supplementary file 2 [file Table_2.docx]

**Supplementary Table 2 Predictive characteristics and birth outcomes for subjects in the second analysis**

| Predictive characteristics | Subjects in the second analysis  (N=3428) | | | | | | | | |
| --- | --- | --- | --- | --- | --- | --- | --- | --- | --- |
|  | Preterm birth | | Low Apgar score | | Birthweight | | | Induction | |
|  | Yes  (N=211) | No  (N=3217) | >=7  (N=24) | < 7  (N=3404) | Low birthweight  (N=147) | Normal  (N=3083) | Macrosomia  (N=198) | Yes  (N=1057) | No  (N=2371) |
| Age n(%) |  |  |  |  |  |  |  |  |  |
| < 30 | 70  (33.2%) | 1514  (47.1%) | 7  (29.2%) | 1577  (46.3%) | 55  (37.4%) | 1446  (46.9%) | 83  (41.9%) | 546  (51.7%) | 1038  (43.8%) |
| 30-39 | 138  (65.4%) | 1665  (51.8%) | 17  (70.8%) | 1786  (52.5%) | 91  (61.9%) | 1602  (52.0%) | 110  (55.6%) | 504  (47.7%) | 1299  (54.8%) |
| >=40 | 3  (1.4%) | 38  (1.2%) | 0  (0%) | 41  (1.2%) | 1  (0.7%) | 35  (1.1%) | 5  (2.5%) | 7  (0.7%) | 34  (1.4%) |
| Ethnicity n(%) |  |  |  |  |  |  |  |  |  |
| Han | 206  (97.6%) | 3146  (97.8%) | 23  (95.8%) | 3329  (97.8%) | 141  (95.9%) | 3021  (98.0%) | 190  (96.0%) | 1031  (97.5%) | 2321  (97.9%) |
| Others | 5  (2.4%) | 71  (2.2%) | 1  (4.2%) | 75  (2.2%) | 6  (4.1%) | 62  (2.0%) | 8  (4.0%) | 26  (2.5%) | 50  (2.1%) |
| Occupation n(%) |  |  |  |  |  |  |  |  |  |
| Company staff | 169  (80.1%) | 2545  (79.1%) | 18  (75.0%) | 2696  (79.2%) | 121  (82.3%) | 2443  (79.2%) | 150  (75.8%) | 852  (80.6%) | 1862  (78.5%) |
| Other occupations | 23  (10.9%) | 432  (13.4%) | 5  (20.8%) | 450  (13.2%) | 14  (9.5%) | 411  (13.3%) | 30  (15.2%) | 143  (13.5%) | 312  (13.2%) |
| Unemployed | 19  (9.0%) | 240  (7.5%) | 1  (4.2%) | 258  (7.6%) | 12  (8.2%) | 229  (7.4%) | 18  (9.1%) | 62  (5.9%) | 197  (8.3%) |
| Gravidity n(%) |  |  |  |  |  |  |  |  |  |
| 1 | 103  (48.8%) | 1673  (52.0%) | 11  (45.8%) | 1765  (51.9%) | 79  (53.7%) | 1598  (51.8%) | 99  (50.0%) | 621  (58.8%) | 1155  (48.7%) |
| >1 | 108  (51.2%) | 1544  (48.0%) | 13  (54.2%) | 1639  (48.1%) | 68  (46.3%) | 1485  (48.2%) | 99  (50.0%) | 436  (41.2%) | 1216  (51.3%) |
| Parity n(%) |  |  |  |  |  |  |  |  |  |
| 1 | 169  (80.1%) | 2713  (84.3%) | 20  (83.3%) | 2862  (84.1%) | 125  (85.0%) | 2599  (84.3%) | 158  (79.8%) | 979  (92.6%) | 1903  (80.3%) |
| >1 | 42  (19.9%) | 504  (15.7%) | 4  (16.7%) | 542  (15.9%) | 22  (15.0%) | 484  (15.7%) | 40  (20.2%) | 78  (7.4%) | 468  (19.7%) |
| Cesarean scar uterus n(%) | 23  (10.9%) | 270  (8.4%) | 1  (4.2%) | 292  (8.6%) | 12  (8.2%) | 266  (8.6%) | 15  (7.6%) | 22  (2.1%) | 271  (11.4%) |
| Gestational diabetes n(%) | 53  (25.1%) | 439  (13.6%) | 5  (20.8%) | 487  (14.3%) | 32  (21.8%) | 423  (13.7%) | 37  (18.7%) | 155  (14.7%) | 337  (14.2%) |
| Gestational hypertension n(%) | 11  (5.2%) | 97  (3.0%) | 1  (4.2%) | 107  (3.1%) | 7  (4.8%) | 95  (3.1%) | 6  (3.0%) | 41  (3.9%) | 67  (2.8%) |
| Preeclampsia n(%) | 4  (1.9%) | 41  (1.3%) | 1  (4.2%) | 44  (1.3%) | 2  (1.4%) | 42  (1.4%) | 1  (0.5%) | 19  (1.8%) | 26  (1.1%) |
| Fever n(%) | 3  (1.4%) | 302  (9.4%) | 6  (25%) | 299  (8.8%) | 3  (2.0%) | 284  (9.2%) | 18  (9.1%) | 165  (15.6%) | 140  (5.9%) |
| Renal disease n(%) | 1  (0.5%) | 19  (0.6%) | 0  (0%) | 20  (0.6%) | 1  (0.7%) | 18  (0.6%) | 1  (0.5%) | 4  (0.4%) | 16  (0.7%) |
| Placenta previa n(%) | 11  (5.2%) | 13  (0.4%) | 0  (0%) | 24  (0.7%) | 6  (4.1%) | 18  (0.6%) | 0  (0%) | 2  (0.2%) | 22  (0.9%) |
| Number of fetus n(%) |  |  |  |  |  |  |  |  |  |
| 1 | 150  (71.1%) | 3172  (98.6%) | 24  (100%) | 3298  (96.9%) | 99  (67.3%) | 3025  (98.1%) | 198  (100%) | 1055  (99.8%) | 2267  (95.6%) |
| >1 | 61  (28.9%) | 45  (1.4%) | 0  (0%) | 106  (3.1%) | 48  (32.7%) | 58  (1.9%) | 0  (0%) | 2  (0.2%) | 104  (4.4%) |
| TSH (mlU/L) mean(SD) |  |  |  |  |  |  |  |  |  |
| Preconception | 1.87  (1.62) | 2.11  (2.86) | 2.57  (2.08) | 2.09  (2.81) | 1.84  (1.70) | 2.07  (1.87) | 2.58  (8.90) | 2.18  (2.40) | 2.05  (2.97) |
| Post-conception | 1.66  (3.01) | 1.83  (1.66) | 3.51  (8.30) | 1.80  (1.63) | 1.77  (3.54) | 1.82  (1.66) | 1.73  (1.44) | 1.88  (1.34) | 1.79  (1.93) |
| Abnormal preconception TSH n(%) | 106  (50.2%) | 1366  (42.5%) | 13  (54.2%) | 1459  (42.9%) | 77  (52.4%) | 1317  (42.7%) | 78  (39.4%) | 472  (44.7%) | 1000  (42.2%) |
| Not well controlled TSH n(%) | 121  (57.3%) | 1596  (49.6%) | 15  (62.5%) | 1702  (50.0%) | 87  (59.2%) | 1540  (50.0%) | 90  (45.5%) | 549  (51.9%) | 1168  (49.3%) |
