# Supplementary material for: Quantifying the Impacts of Pre- and Post-Conception TSH Levels on Birth Outcomes: An Examination of Different Machine Learning Models
Source: Front Endocrinol (Lausanne). 2021 Oct 29;12:755364. doi: 10.3389/fendo.2021.755364 (PMC8586450; doi:10.3389/fendo.2021.755364)
Supplement: Supplementary file 3 [file Table_3.docx]

**Supplementary Table 3 Detailed model metrics of synthetic data from subjects in the first analysis**

| Predictive models | Metrics | Preterm Birth | Low Apgar Score^a^ | Birthweight^b^ | Induction |
| --- | --- | --- | --- | --- | --- |
| Logistic model | Accuracy | 64.1% | 75.8% | 47.5% | 59.9% |
|  | Precision | 73.3% | 78.4% | 50.4% | 57.1% |
|  | Recall | 44.7% | 71.9% | 47.6% | 85.6% |
|  | F1 score | 55.5% | 75.0% | 47.6% | 68.5% |
|  | AUC | 64.2% | 75.8% |  | 59.5% |
| Random forest model | Accuracy | 65.5% | 77.0% | 48.4% | 60.3% |
|  | Precision | 75.7% | 77.1% | 60.0% | 57.2% |
|  | Recall | 45.8% | 77.7% | 48.7% | 86.3% |
|  | F1 score | 57.1% | 77.4% | 47.8% | 68.8% |
|  | AUC | 65.6% | 77.0% |  | 59.9% |
| XGBoost model | Accuracy | 65.8% | 81.0% | 50.4% | 59.8% |
|  | Precision | 74.4% | 76.9% | 52.6% | 57.7% |
|  | Recall | 48.2% | 89.1% | 50.5% | 77.9% |
|  | F1 score | 58.5% | 82.6% | 50.7% | 66.3% |
|  | AUC | 65.8% | 80.9% |  | 59.5% |
| Multilayer neural network | Accuracy | 63.8% | 74.9% | 45.0% | 59.3% |
|  | Precision | 72.9% | 80.2% | 48.8% | 59.4% |
|  | Recall | 44.1% | 66.9% | 45.3% | 63.3% |
|  | F1 score | 55.0% | 72.9% | 42.7% | 61.2% |
|  | AUC | 63.4% | 75.0% |  | 59.3% |

** 18 dummy predictive features were adjusted in four models*

*a. 5 more variables on delivery process were adjusted in the predictive model of low Apgar score. The 5 extra variables were fetal position, neonatal injury during delivery, delivery method, lateral episiotomy and vaginal midwifery.*

*b. Model performance of birthweight was assessed with macro F1 score instead of AUC.*
