# Supplementary material for: Quantifying the Impacts of Pre- and Post-Conception TSH Levels on Birth Outcomes: An Examination of Different Machine Learning Models
Source: Front Endocrinol (Lausanne). 2021 Oct 29;12:755364. doi: 10.3389/fendo.2021.755364 (PMC8586450; doi:10.3389/fendo.2021.755364)
Supplement: Supplementary file 4 [file Table_4.docx]

**Supplementary Table 4 Model performance for predicting birthweight with different two TSH scenarios in the second analysis**

| **TSH scenarios** | **Metrics** | **Abnormal preconception TSH** | **Not-well-controlled TSH** |
| --- | --- | --- | --- |
| Logistic model | Accuracy | 48.5% | 48.5% |
|  | Precision | 49.8% | 49.3% |
|  | Recall | 48.5% | 48.5% |
|  | F1 Score | 48.5% | 48.6% |
| Random forest model | Accuracy | 52.0% | 52.8% |
|  | Precision | 56.0% | 56.9% |
|  | Recall | 52.0% | 52.8% |
|  | F1 Score | 50.9% | 51.8% |
| XGBoost model | Accuracy | 55.6% | 54.5% |
|  | Precision | 57.6% | 54.5% |
|  | Recall | 55.5% | 54.4% |
|  | F1 Score | 54.3% | 53.9% |
| Multilayer neural network | Accuracy | 48.9% | 45.4% |
|  | Precision | 53.7% | 49.0% |
|  | Recall | 48.9% | 45.5% |
|  | F1 Score | 47.9% | 41.8% |

** 18 dummy predictive features were adjusted in four models*
